# The effects of supplementing maternal and infant diets with lipid-based nutrient supplements on physical activity and sedentary behaviour at preschool age in Ghana

**DOI:** 10.1017/S0007114519001636

**Published:** 2019-09-16

**Authors:** Maku E. Ocansey, Anna Pulakka, Seth Adu-Afarwuah, Rebecca R. Young, Sika M. Kumordzie, Harriet Okronipa, Brietta M. Oaks, Kathryn G. Dewey, Elizabeth L. Prado

**Affiliations:** 1 Program in International and Community Nutrition, Department of Nutrition, University of California, Davis, CA 95616, USA; 2 Department of Public Health, University of Turku and Turku University Hospital, 20014 Turku, Finland; 3 Department of Nutrition and Food Science, University of Ghana, Legon 20520, Ghana; 4 Department of Nutrition and Food Sciences, University of Rhode Island, Kingston, RI 02881, USA

**Keywords:** Lipid-based nutrient supplements, Physical activity, Accelerometers, Preschool-age children, Ghana

## Abstract

Evidence on whether nutritional supplementation affects physical activity (PA) during early childhood is limited. We examined the long-term effects of lipid-based nutrient supplements (LNS) on total PA, moderate-to-vigorous PA (MVPA) and sedentary behaviour (SB) of children at 4–6 years using an accelerometer for 1 week. Their mothers were enrolled in the International Lipid-based Nutrient Supplement-DYAD randomised controlled trial in Ghana, assigned to daily LNS or multiple micronutrients (MMN) during pregnancy through 6 months postpartum or Fe and folic acid (IFA) during pregnancy and placebo for 6 months postpartum. From 6 to 18 months, children in the LNS group received LNS; the other two groups received no supplements. Analysis was done with intention to treat comparing two groups: LNS *v*. non-LNS (MMN+ IFA). Of the sub-sample of 375 children fitted with accelerometers, 353 provided sufficient data. Median vector magnitude (VM) count was 1374 (interquartile range (IQR) 309), and percentages of time in MVPA and SB were 4·8 (IQR 2) and 31 (IQR 8) %, respectively. The LNS group (*n* 129) had lower VM (difference in mean −73 (95 % CI −20, −126), *P* = 0·007) and spent more time in SB (LNS *v*. non-LNS: 32·3 *v*. 30·5 %, *P* = 0·020) than the non-LNS group (*n* 224) but did not differ in MVPA (4·4 *v*. 4·7 %, *P* = 0·198). Contrary to expectations, provision of LNS in early life slightly reduced the total PA and increased the time in SB but did not affect time in MVPA. Given reduced social-emotional difficulties in the LNS group previously reported, including hyperactivity, one possible explanation is less restless movement in the LNS group.

The foundations for health behaviours such as physical activity (PA) and sedentary behaviour (SB) are laid from a very young age^(1)^ and have long-term consequences for health and development. Increased PA and reduced SB in childhood have been associated with a reduced risk for high adiposity^(2)^ and accelerated weight gain^(3)^ and reduced cardiometabolic risk factors including insulin resistance, diastolic blood pressure and LDL-cholesterol^(4–6)^, as well as improved musculoskeletal health^(6,7)^, psychosocial well-being^(8)^, motor skills^(9)^ and cognitive function and academic achievement^(10,11)^. Accurate and reliable measurement of PA of a population is, therefore, important for monitoring health as well as the effectiveness of interventions^(12)^.

Undernourished children have been shown to be less physically active than their well-nourished counterparts^(13)^, and increases in activity levels have been shown to correspond with improvements in nutritional status during childhood^(14)^. In response to low dietary intakes of energy and protein, undernourished children exhibit behavioural changes including reduced PA and exploration in their environment^(15)^, which may persist into adolescence^(13)^. Deficiencies in micronutrients, particularly Fe^(16–18)^ and Zn^(19,20)^, are associated with reduced PA in early childhood; however, evidence for the effects of micronutrient supplementation during early life on behavioural PA in randomised trials has been mixed^(19–28)^, and only two such studies^(25,26)^ have used accelerometers to measure PA outcomes. Accelerometers provide an objective, practical, accurate and reliable measure of the amount and intensity of PA and SB among preschool children, compared with indirect methods such as questionnaires or interviews^(29,30)^.

Unlike multiple micronutrient (MMN) supplements, lipid-based nutrient supplements (LNS) are energy-dense food-based supplements providing a full set of macro- and micronutrients, including essential fatty acids, protein and MMN to support healthy child growth and development. In the present study, we investigated the long-term effects of maternal LNS during pregnancy and 6 months postpartum and infant supplementation from 6 to 18 months of age on PA and SB at age 4–6 years measured using accelerometers. We hypothesised that LNS supplementation during pregnancy, postpartum and infancy would increase PA and lower SB at 4–6 years compared with maternal Fe and folic acid (IFA) supplementation during pregnancy only, or MMN supplementation during pregnancy and through 6 months postpartum.

## Methods

### Study design

*Original trial*. The International Lipid-based Nutrient Supplements (iLiNS)-DYAD Ghana study was a randomised, partially double-blind, controlled trial conducted between 2009 and 2014 in two semi-urban districts (Yilo and Manya Krobo) of the Eastern region of Ghana, located about 70 km north of the capital, Accra. The overall objective of the iLiNS-DYAD Ghana trial was to test the efficacy of three types of nutrient supplements for preventing malnutrition in pregnant and lactating women and their infants. A detailed description of the design and methods of the original trial (ClinicalTrials.gov; identifier NCT00970866) has been published elsewhere^(31)^. Briefly, pregnant women aged ≥18 years attending antenatal clinics in four health facilities in the area were recruited to participate. Women were excluded if they had HIV infection, asthma, epilepsy, tuberculosis, any malignancy, or known milk or peanut allergies. Women who resided outside the study area or were >20 weeks of gestational age before completion of the enrolment process were also excluded.

A total of 1320 pregnant women were randomly assigned to 60 mg/d Fe and 400 μg/d folic acid until delivery and placebo thereafter, and no supplementation for infants (IFA group: *n* 441); or MMN containing 20 mg/d Fe until 6 months postpartum and no supplementation for infants (MMN group: *n* 439); or small-quantity LNS (LNS group: *n* 440) containing 20 mg/d Fe until 6 months postpartum, and LNS for infants from 6 to 18 months of age^(31)^.

*Follow-up study*. The sub-study reported here was part of a larger follow-up study of children whose mothers were enrolled in the iLiNS-DYAD Ghana trial. The follow-up study took place from January to December 2016, when children were 4–6 years of age. All children born to pregnant women who had been randomised to one of the three iLiNS-DYAD trial groups were eligible for re-enrolment in the follow-up study irrespective of whether the mother was lost to follow-up before or after delivery. Children were excluded if the mother or caregiver was unwilling to consent to the child’s participation or they were not residing within the study site (Yilo and Manya Krobo districts) or surrounding towns at the time of study. Participation of children was by parental or caregiver written or thumb-printed informed consent after trained data collectors visited the homes to explain the study. Ethical approval for the follow-up study was obtained from the ethics committee of the University of California, Davis, the Ethics Committee for the College of Basic and Applied Sciences of the University of Ghana and the Ghana Health Service Ethical Review Committee.

### Random selection of sub-study participants

**Fig. 1.**
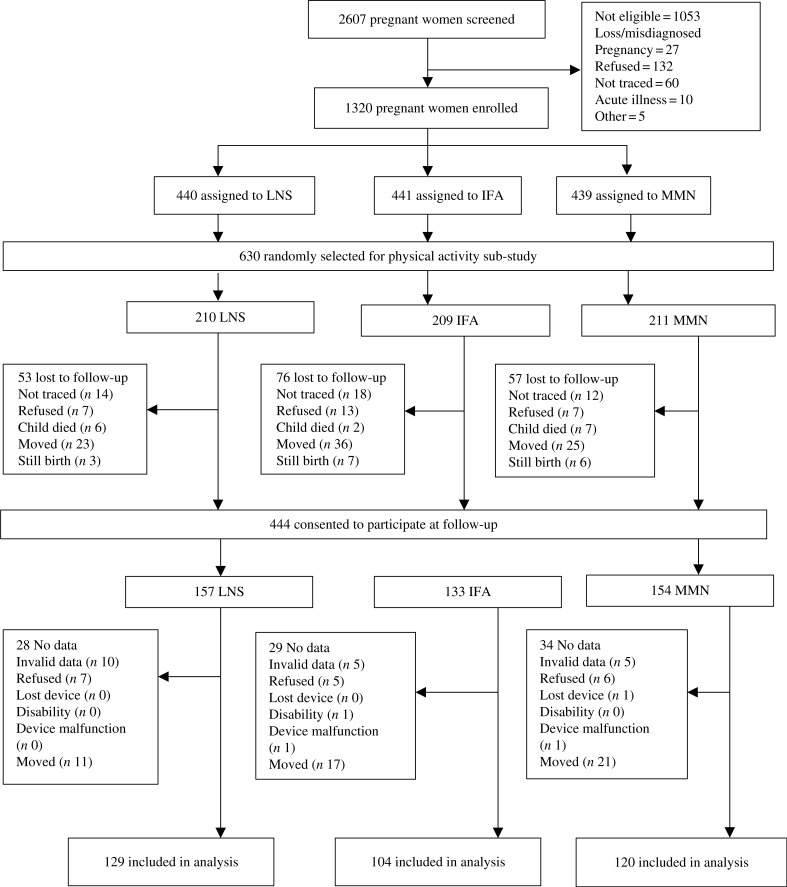
Study profile showing infants whose mothers were enrolled into the trial, and the reasons some infants were lost to follow-up. LNS, lipid-based nutrient supplement; IFA, iron and folic acid; MMN, multiple micronutrient. In the LNS group, women received 20 g LNS daily during pregnancy and 6 months postpartum. Infants received 20 g LNS daily from 6–18 months of age. In the non-LNS group, women received either IFA during pregnancy and placebo for 6 months postpartum or MMN capsules during pregnancy and 6 months postpartum. Infants did not receive any supplement. Groups shown are based on supplements women received at enrolment.

For this sub-study, the sample size was based on detecting an effect size of 0·33 standard deviations or greater in mean vector magnitude (VM) counts per min, which was the primary outcome. We calculated a required sample size of 396 participants (132 per intervention group) to detect the differences between the three groups, given a power of 80 and 5 % level of significance. Accounting for an attrition rate of 17·5 %, a sub-sample of 480 children of all eligible participants was initially randomly selected by a statistician who was not involved in data collection, using a blocked randomisation list. The high rate of participant migration from the study area resulted in higher attrition than expected; thus, an additional 150 children were randomly selected to participate, making a total of 630 children (see Fig. 1).

### Measurement and calculation of variables

The primary outcome was the mean VM counts per min during waking hours. The secondary outcomes were the percentage of (i) waking hours spent in moderate-to-vigorous PA (MVPA), (ii) waking hours spent in SB and (iii) children who met the minimum activity recommendation of ≥60 min of MVPA/d. Each participant was outfitted with a single wGT3X-BT ActiGraph accelerometer to monitor and measure PA over a 1-week period, day and night. The devices were distributed during home visits by trained data collectors blinded to the intervention group. The small accelerometers were fitted to an elastic belt and fastened to the child’s right hip during a home visit the day before the first day of measurement, and data collectors made routine home visits mid-weekly to ensure that they were correctly placed. The devices were programmed to record activity measurements for seven consecutive days, starting at 05.00 hours on the first day and ending at 21.00 hours on the last day. Parents and caregivers were instructed that the children should wear the device continuously day and night without removing it unless they experienced major discomfort. Data collectors visited participants’ homes on or after the 8th day to remove devices after which recorded data were downloaded.

Socio-demographic information was collected at enrolment into the original trial, including maternal education, maternal age, nulliparity and a household assets score, constructed based on ownership of a set of assets (radio, television (TV), etc.), lighting source, drinking water supply, sanitation facilities and flooring materials. The household asset score was created using principal components analysis and had a mean of 0 and an SD of 1, with higher values representing higher socio-economic status^(32)^.

Two trained and standardised anthropometrists measured children’s height to the nearest 0·1 cm using a Seca stadiometer and weight to the nearest 50 g using a Seca scale. Weight-for-age *z*-score and BMI-for-age *z*-scores were calculated based on standard procedures^(33)^. Fat mass percentage was determined using the ^2^H dilution method. Details of this method have been previously reported^(34)^.

Information on preschool attendance and the month of data collection was also collected at follow-up by means of a questionnaire. Based on rainfall patterns in the study area, the months of November to April were classified as the dry season and May to October as the wet season. Parents or caregivers provided the name and location of the preschools attended by the participating child, and a brief survey was administered to heads of preschools to identify the type of instruction (e.g. free play or structured instruction), and the number of hours of instruction provided for different age groups in the school.

Cognitive, motor and social–emotional assessments were conducted at follow-up using standard tests that were adapted and validated for this population. Details of the tests have been described elsewhere^(35)^. We assessed the amount and quality of developmental nurturing and stimulation available to children in the home environment at follow-up using the Early Childhood version of the Home Observation for the Measurement of the Environment Inventory^(36)^, which we adapted to the local context.

### Data reduction and statistical analysis

All data reduction was done with ActiLife software (ActiGraph LLC). Each child’s mean VM counts per min was calculated as the square root of the sum of squared activity counts of three axes using the ActiLife software version 6.13.1. We took the mean of the mean counts per min of each day over all the valid days within the week for each child. A day was considered valid when there was a minimum of 10 h of wake time accelerometer data. Only children with ≥4 valid days (minimum of 2 weekdays and 1 weekend day) of data were included in the analyses. Activity counts were stored in 60-s intervals or epochs. We defined and excluded sleep time based on the sleep prediction equation developed by Sadeh *et al*.^(37)^ and the sleep period detection option created by Tudor-Locke *et al*.^(38)^. Non-wear time, defined as strings (consecutive periods) of ≥20 min of zero counts, was excluded^(39)^.

The percentage of time spent in MVPA, light PA and SB, was calculated using cut-offs previously validated by Evenson and colleagues^(40)^ for children aged 5–8 years. Percentage of time spent in MVPA (vertical axis activity counts of ≥2296 counts per min) per day was averaged over all valid days within a 7-d period and the average value (per child) was used in the analysis. The percentages of time spent in light PA and SB were calculated based on vertical axis activity counts between 100 and 2296 counts per min and ≤100 counts per min, respectively, and were averaged over all valid days per child. Children whose mean time in MVPA over all valid days was ≥60 min/d were considered to have met minimum PA levels. We used the guidelines of the US National Association for Sports and Physical Education^(41)^ due to the lack of local PA guidelines for children in Ghana^(42)^ or other low- to middle-income countries.

We posted a statistical analysis plan with prespecified potential covariates and effect modifiers to the project website (www.ilins.org) before study investigators were unblinded to children’s intervention group assignments. All analyses were conducted using SAS version 9.4 (SAS Institute). We examined all continuous data by univariate analysis to identify outliers. The percentage of time spent in MVPA was not normally distributed; therefore, it was log-transformed.

We examined whether children in the three intervention groups were different with regard to a number of baseline and follow-up characteristics using ANOVA for continuous variables and χ^2^ tests for binary variables. To examine selection to the study sample, we also compared differences in baseline characteristics between the children included in this analysis and children enrolled in the original trial but not included in this analysis. Among the 630 children randomly selected to be included in this sub-study, we examined whether selected baseline and follow-up characteristics were different between children analysed in the study and those lost to follow-up. For any characteristics that were significantly different, we tested whether they were significantly associated with the primary or secondary outcomes. Subsequently, we imputed values for the outcome variables for the children who were lost to follow-up, based on baseline and other characteristics that were associated with the PA outcomes. We imputed five sets of values for each PA outcome using SAS PROC MI. In a sensitivity analysis, we examined the difference between the intervention groups using the imputed missing values to see if the results differed from the results including only the children with non-missing PA data.

In the analysis of the primary and secondary outcomes, we first tested the null hypothesis of no difference between the three intervention groups using ANCOVA for continuous outcomes and logistic regression for binary outcomes. For each primary or secondary outcome, we performed post hoc pairwise comparisons of the three intervention groups using Tukey–Kramer adjustment, and whenever there was no significant difference between the IFA and MMN groups (i.e. *P* not less than 0·05), we combined the groups into a single control (non-LNS) group to estimate two-group comparisons (LNS *v*. non-LNS).

We compared groups using two statistical models. The first model was adjusted only for child age at follow-up (model 1). The second model was additionally adjusted for any of the following prespecified background or follow-up variables that were significantly associated at the *P* < 0·1 level with the outcome of interest in correlation analysis: maternal age, maternal education, household assets score, sex, parity, preschool attendance and season of data collection. For any potential covariates collected at follow-up, we first checked whether they were no differences between the groups before including them in the model since they could be potential mediators.

We performed additional exploratory analyses based on the same statistical models described previously to assess the differences between the groups separately for weekdays (from Monday to Friday) and weekends (Saturday or Sunday), and to examine the effect of the intervention on light PA and the highest and lowest quartiles of the mean VM counts per min in order to further understand the pattern of results. Additionally, we examined associations between PA indicators and children’s percent body fat, social–emotional development and ownership of TV in the home.

## Results

### Follow-up and characteristics of the study sample

The total number of potential participants randomly selected for this sub-study was 630: 209 in the IFA group, 211 in the MMN group and 210 in the LNS group. We obtained parental or caregiver consent for the follow-up study for 444 of these 630. Of the 186 not consented, we excluded sixteen stillbirths and fifteen child deaths that occurred before or after ending the original trial. Of the remaining 155, we were unable to trace forty-four children, eighty-four migrated out of the study area and twenty-seven refused participation. We collected accelerometer data for 375 out of the 444 consented children. Data were not collected for sixty-nine children because caregiver consent for the child to wear the accelerometer was declined (*n* 18), the child had a disability in their limbs (*n* 1), the child lost the device (*n* 1) or the child had moved out of the study area when it was time to wear the accelerometer (*n* 49). Data from twenty-two children (5·9 % of 375) were excluded because the information collected was insufficient for the analysis based on the criteria described in the methods section (*n* 20) or there was a device malfunction (*n* 2). Thus, data for 353 children were included in these analyses: 129 in the LNS group, 104 in the IFA group and 120 in the MMN group. We found that baseline maternal age and parity, as well as child motor development score at 18 months, were significantly different between the 353 children included in the analysis and the 277 who were not included (Supplementary Table S1). Nulliparity was associated with greater time being sedentary (*r* 0·11; *P* = 0·033) but not baseline maternal age. Of the 630 children randomly selected for this sub-study, the proportion of children lost to follow-up was greater in the IFA group (50·2 %) compared with the LNS (38·6 %, *P* = 0·016) and MMN (43·1 %, *P* = 0·145) groups.

Table 1 shows the baseline characteristics of children with sufficient accelerometer data in the three intervention groups at age 4–6 years. There were no significant differences across intervention groups in any of the four baseline characteristics described. At follow-up, children did not differ in any of the seven characteristics measured, including the number of valid days of accelerometer wear and accelerometer wake wear time (*P* > 0·05). The two-group comparisons are presented in Supplementary Table S2.

**Table 1.** Selected characteristics of women and children by the three intervention groups at baseline and follow-up* (Mean values and standard deviations; percentages and numbers of participants)

	Fe and folic acid (n 104)			Multiple micronutrient (n 120)			Lipid-based nutrient supplement (n 129)		
Variable	Mean	SD	*n*/total *n*	Mean	SD	*n*/total *n*	Mean	SD	*n*/total *n*
Collected at baseline									
Maternal age (years)	27·1	0·5	104	27·3	0·5	120	27·8	0·5	129
Maternal education (years)	7·4	0·4	104	7·4	0·3	120	7·9	0·3	129
Household asset score†	0·07	0·10	104	0·18	0·09	119	−0·02	0·09	125
Nulliparous (%)	27·9		29/104	29·2		35/120	29·5		38/129
Collected at follow-up									
Child male (%)	46·2		48/104	45·0		54/120	43·4		56/129
Age at follow-up (years)	5·2	0·1	104	5·1	0·1	120	5·2	0·1	129
Valid days of accelerometer wear (no.)	6·7	0·1	104	6·8	0·1	120	6·8	0·1	129
Accelerometer wake wear time (h)	14·7	0·1	104	14·6	0·1	120	14·8	0·1	129
Time spent in preschool instruction (h)	5·2	0·1	104	5·3	0·1	120	5·2	0·1	129
Preschool attendance (%)	84·6		88/104	85·8		103/120	86·8		112/129
Season measurement: Wet (%)	73·1		76/104	70·0		84/120	75·2		97/129
Home stimulation score at 4–6 years	27·4	0·5	104	28·1	0·4	119	28·3	0·4	129

* Non-lipid-based nutrient supplement group (control group) was the Fe and folic acid + multiple micronutrient capsule groups.

† Proxy indicator for household socio-economic status constructed for each household based on ownership of a set of assets (radio, television, etc.), lighting source, drinking water supply, sanitation facilities and flooring materials. Household ownership of this set of assets is combined into an index (with a mean of 0 and an SD of 1) using principal components analysis. Higher value represents higher socio-economic status^(31).^

### Effect of the intervention on the primary and secondary activity outcomes

The mean number of days with valid accelerometer data per child was 6·74 (SD 0·65), ranging between 4 and 7 d. The median number of hours per day of activity measurement excluding sleep and non-wear time was 14·7 (interquartile range 12·4, 17·8). We did not find any significant differences between the IFA and MMN groups in the primary outcome or any of the secondary outcomes; therefore, we combined the two groups into a single control group (non-LNS group). We report here the two-group comparisons with analyses performed by intention-to-treat. The three-group comparisons are presented in Supplementary Table S3.

The mean VM accelerometer counts per min and percentage of time spent in SB for the total sample were 1387 (SD 246) and 31 % (SD 6·8), respectively. The geometric mean of percentage of time spent in MVPA for the total sample was 4·6 (95 % CI 4·3, 4·8) %. Intervention group differences are presented in Table 2. When adjusting for child age at follow-up, the LNS intervention group had a significantly lower mean VM accelerometer counts per min than the non-LNS group (*P* = 0·007) as well as a significantly higher average percentage of time spent in SB than the non-LNS group (*P* = 0·020). However, there were no significant differences between the groups in the average percentage of time spent in MVPA (*P* = 0·198). With adjustment for baseline and additional covariates, the differences between groups for VM counts per min (*P* = 0·0006; see footnote to Table 2) and average percentage of time spent in SB (*P* = 0·02 see footnote to Table 2) remained significant, and there were no significant differences between the groups in average percentage of time spent in MVPA (*P* = 0·175; see footnote to Table 2).

**Table 2.** Physical activity at 4–6 years by intervention group* (Mean values or geometric means and 95 % confidence intervals; differences or ratios of geometric means and 95 % confidence intervals; percentages and numbers of participants)

	Treatment group						Comparison between groups			
	LNS (*n* 129)			Non-LNS (*n* 224)			Difference in mean or ratio of the geometric mean	95 % CI	*P* adjusted for child age at -follow-up	*P* adjusted for baseline and other covariates
	Mean	95 % CI	*n*/total *n*	Mean	95 % CI	*n*/total *n*				
VM (counts/min)	1340	1298, 1382		1413	1382, 1445		−73	−126, −20	0·007	0·006†
Time spent in moderate-to-vigorous physical activity‡ (%)	4·4	4·0, 4·7		4·7	4·4, 4.9		1·1	1·0, 1·2	0·198	0·175§
Time spent in sedentary behaviour (%)	32·3	31·1, 33·4		30·5	29.6, 31.4		1.8	0·3, 3.3	0·020	0·020║
Time spent in light physical activity (%)	62·9	61·8, 63.9		64·2	63.4, 65.0		1.4	0.02, 2.7	0·047	0·056¶
Highest quartile of VM (counts/min) (%)	14·7		19/129	31.2		70/224	–		<0·001	<0·001**
Lowest quartile of VM (counts/min) (%)	30·2		39/129	21.9		49/224	–		0·066	0·064††

LNS, lipid-based nutrient supplement; Non-LNS, Fe and folic acid þ multiple micronutrient capsule groups (control group); VM, vector magnitude; IFA, Fe and folic acid; MMN, multiple micronutrients; HOME, Home Observation for the Measurement of the Environment.

* We first tested the null hypothesis of no difference between the three treatment groups and then combined the IFA/MMN groups because there were no significant differences between those two groups.

† Adjusted for child age at follow-up and child sex.

‡ Analysed with log-transformed values. Geometric mean (95 % CI) and ratio of the geometric mean (95 % CI) presented.

§ Adjusted for child age at follow-up, child sex and HOME score.

║ Adjusted for child age at follow-up and nulliparity.

¶ Adjusted for child age at follow-up, nulliparity, child sex and HOME score.

** Adjusted for child age at follow-up, nulliparity, child sex and HOME score.

†† Adjusted for child age at follow-up, nulliparity, child sex and HOME score.

The percentage of the children who reached an average of at least 60 min of MVPA/d, as recommended by US National Association for Sports and Physical Education, was 17·1 % in the LNS group and 24·6 % in the non-LNS group (adjusted for child age *P* = 0·082; adjusted for additional covariates *P* = 0·059).

### Sensitivity analyses

As mentioned above, children lost to follow-up had mothers with younger age and higher parity at baseline and had a lower motor development score at 18 months, compared with children in these analyses. Child motor development score at 18 months was positively associated with all PA outcomes at 4–6 years (*r* < 0·2, *P* < 0·05). We, therefore, performed a sensitivity analysis using the full 630 children randomly selected for the present study with multiple imputations of missing outcome scores. The differences between intervention groups were similar to the results obtained without imputation, though somewhat attenuated: the difference in means was −54 (95 % CI −99, −9·0), *P* = 0·019 for VM count, 1·0 (95 % CI 0·96, 1·1), *P* = 0·339 for time in MVPA and 1·20 (95 % CI 0·05, 2·4), *P* = 0·060 for time in SB.

### Exploratory analyses

Although we had not prespecified the percentage of time in light PA or the highest quartile of VM counts per min as outcomes, we conducted exploratory analyses examining these variables in order to better understand the pattern of results (also shown in Table 2). Children in the LNS group spent a significantly lower percentage of time in the light PA than in the non-LNS group (adjusted for child age *P* = 0·047). A lower proportion of children in the LNS group were in the highest quartile of VM counts per min (14·7 %) compared with the non-LNS group (31·2 %, *P* = 0·0008). A higher proportion of children in the LNS group were in the lowest quartile of VM counts per min (31·2 %) compared with the non-LNS group (21·9 %), but this difference was not statistically significant (adjusted for child age *P* = 0·066).

Additional analyses of children’s activity separately for weekdays and for weekends showed similar results for the primary and secondary outcomes. During both weekdays and weekends, children in the LNS group had significantly lower VM counts per min and a higher average percentage of time spent in SB than in the non-LNS children, but there were no significant differences in MVPA (see Supplementary Tables S4 and S5).

The unadjusted anthropometric parameters and body composition results of children in this sample are shown in Supplementary Table S6. There were no significant group differences between children in weight-for-age *z*-score (*P* = 0·243), BMI-for-age *z*-scores (*P* = 0·840) or fat mass percentage (*P* = 0·860). Preschool attendance in this sample was similar between the groups in both three- (see Table 1) and two-group comparisons (LNS 86·8 %; non-LNS 85·3 %; *P* = 0·687), and the number of hours of instruction per day children received in school (*v*. time in free play) was similar in the three-group (see Table 1) and two-group comparisons (LNS = 5·2; non-LNS = 5·3; *P* = 0·364). TV ownership did not differ by intervention group (LNS = 86·8 %; non-LNS = 84·8 %; *P* = 0·607).

## Discussion

In this follow-up study to assess the long-term effects of a randomised controlled trial of maternal and infant supplementation with small-quantity LNS on the PA and SB of children in Ghana, we found significant but relatively small differences between LNS-supplemented and non-LNS children at preschool age, in the opposite direction of what was expected. LNS-supplemented children had lower mean accelerometer counts (73 VM counts per min less out of a mean of approximately 1400 counts per min) compared with non-LNS children. They spent less time in light PA (average of 1·4 percentage points lower) and more time in SB (average of 1·8 percentage points more) but did not differ significantly from non-LNS children in time spent in MVPA.

Our study is the first, to our knowledge, to follow up children who received LNS supplementation in the prenatal and postnatal periods and to examine the effects on PA in the long term. Two randomised controlled trials in Malawi assessed accelerometry-derived PA in 18-month-old infants immediately after LNS supplementation during either the postnatal period or both the prenatal and postnatal periods. In the postnatal supplementation trial, there were no significant effects of 10- to 40-g quantities/d of LNS provided from 6 to 18 months of age on PA outcomes at 18 months^(25)^. The prenatal and postnatal supplementation study in Malawi^(26)^ was conducted in parallel with the trial in Ghana described here and had the same intervention groups and design. In Malawi, no differences were found between the LNS, MMN and IFA groups in any PA outcomes measured at 18 months. Findings from these two studies in Malawi are not consistent with the negative effects we found on mean accelerometer counts and percentage of time spent in SB in the LNS group at preschool age, but consistent with the null effects of LNS supplementation on MVPA levels. Our findings of no effect of supplementation on MVPA are also consistent with the findings from two other studies that did not find effects of supplementation on PA in 24-month-old children or school-age children^(24,27)^.

Our findings are not consistent with five previous randomised controlled trials that found positive effects of nutritional supplementation during infancy or early childhood on physical or motor activity providing MMN supplements in addition to macronutrients^(22,23,28)^ or Zn^(19,20)^ for a period of 4–12 months. In these studies, PA was assessed by direct observation^(22,23)^, time sampling observations^(19,20)^ or parental reports based on PA questionnaires^(28)^, which are more susceptible to bias (including participant’s reactivity to the observer) compared with 7-d accelerometer wear used in our study, which is a more objective measure. Furthermore, these studies showing positive effects of nutritional supplementation on PA were conducted among children at risk of nutritional problems including wasting, stunting, Fe-deficiency anaemia, Zn deficiency and diarrhoea. Undernourished or sick children could have reduced energy leading to less participation in PA and may therefore be more likely to increase PA after micro- and/or macronutrient supplementation. In our study, we enrolled mothers during pregnancy and did not target children with poor nutritional status. Finally, all of the aforementioned studies measured PA or motor activity immediately after supplementation while we measured PA 2–4 years later. The substantial time difference could possibly account for the differences in results in our study and the other studies.

We explored several potential explanations for our results. First, we examined whether there was a higher prevalence of obesity or overweight in children in the LNS group. Obesity or overweight could result in the reduction of PA, and SB has been associated with obesity risk in childhood^(43)^. However, there were no significant differences in BMI-for-age *z*-scores, percentage of fat mass, or weight-for-age *z*-score between the groups (see Supplementary Table S5).

Second, we examined whether the proportion of children attending preschool or the number of hours per day in preschool differed between groups, which might affect PA. The majority of children (86 %) attended preschool, and on average, children attended preschool for about 7·3 h/d, which is about half the median accelerometer wear time in the present study (14·7 h/d). A school environment providing little time for free play could translate into greater percentage of time in SB. However, the proportion of children attending preschool was similar between groups (*P* = 0·687), and there was no group difference in the number of hours of instruction (as opposed to hours in free play) received in preschool (*P* = 0·364). Moreover, we found lower activity in the LNS group on both weekends and weekdays, supporting the conclusion that preschool attendance is unrelated to differences in activity between intervention groups in this sample.

Third, we considered whether TV viewing, one of the most common indicators of SB among children and youth, may have accounted for the differences in activity between groups. In this sample, greater than 96 % of households owned TV sets, and TV ownership did not differ by the intervention group (*P* = 0·607). We do not have data on hours spent per day watching TV.

Finally, we explored whether the difference in PA between the LNS and non-LNS groups could be related to the children’s social-emotional development and behavioural problems. In the larger iLiNS-DYAD Ghana follow-up cohort from which the present study sample was selected, children in the LNS group had a lower score for behavioural problems compared with the non-LNS group based on the Strengths and Difficulties Questionnaire^(35)^. The total difficulties score of the Strengths and Difficulties Questionnaire is made up of four sub-scales: emotional symptoms, conduct problems, hyperactivity and peer problems^(44)^. Both the hyperactivity sub-scale and the overall social-emotional difficulties score were positively associated with mean accelerometer counts per min in this sample (*P* ≤ 0·05) and negatively correlated with the average percentage of time spent in SB, although the latter relationship was not significantly different (*P* ≤ 0·08) (Supplementary Table S7). Thus, overall, children with lower social-emotional difficulties were less physically active, and the LNS group showed both lower social-emotional difficulties and lower PA than the non-LNS group. The possibility that differences in social-emotional development account for differences in PA between the LNS and non-LNS groups is supported by the result that the difference between groups was primarily in light PA, which could include behaviours such as restless movement associated with hyperactivity, whereas there were no differences between groups in MVPA. Additional evidence supporting this explanation is that the group difference in mean accelerometer counts was primarily in the upper end of the distribution: 15 % of children in the LNS group were in the highest quartile of activity, compared with 31 % of children in the non-LNS group (OR 0·38 (95 % CI 0·22, 0·67), *P* < 0·001), whereas the proportions in the lowest quartile of activity were 31·2 *v*. 21·9 %, respectively (OR 1·59 (95 % CI 0·97, 2·62), *P* = 0·066). Of the potential explanations we explored, this final possibility seems to be the most plausible.

Children in our sample spent on average 264 min/d (equivalent to 31 % of waking h/d) being sedentary. This is within the range of 23 to 95 % of wear time in SB reported in a review of 30 studies based on accelerometry in preschool-aged children in developed countries^(45)^. MVPA in this sample averaged 43 min/d (equivalent to 4·4 % of waking h/d), which is slightly lower than the US recommendation of at least 60 min/d for this age group^(41,46)^; about 22 % of children met this recommendation. Our findings are similar to those of Bornstein and colleagues^(47)^ who reported an average of 5·5 % wear time in MVPA/d in a meta-analysis of 29 studies in developed countries (*n* 6309) using accelerometry-derived PA in preschoolers, and, also within the range of 1·7–41·2 % wear time in MVPA/d reported in a review of 40 studies on preschool children^(45)^. However, comparisons across studies need to be made cautiously because there are no globally accepted cut points to classify PA levels in children and differences in cut points^(45,48)^ and/or epoch lengths^(49)^ used could over or underestimate MVPA.

Generally, parents consider preschoolers to be highly active^(50)^, but there is growing evidence that the proportion of children meeting PA and SB recommendations is low, as seen with our cohort and several studies showing insufficient amounts of PA^(45,47)^ and greater sedentary time than recommended for their age group^(51–54)^. A call for action is, therefore, needed to reverse this trend among young children and youth for better health outcomes in the long term. We found a negative association of both the mean VM counts per min and percentage of time spent in MVPA with the percentage of body fat of children (Supplementary Table S8). This finding is consistent with previous studies showing associations between higher PA and decreased adiposity among children and adolescents^(2,5,6)^ and emphasises the importance of PA for body composition. Care must be taken to ensure that the low activity levels among preschool children in this population do not persist into adulthood, thereby increasing the risk of obesity and related diseases.

Strengths of the present study include the randomisation of participants to intervention groups at baseline and the blinding of PA data collectors and analysts to intervention groups. We used an objective measurement of PA and SB (hip-worn accelerometers) that is a standardised and widely used method for assessing PA in free-living children. We implemented standard procedures during data processing to exclude unreliable data and used validated thresholds specifically for the preschool age group to define PA intensities. The longitudinal nature of our study design enabled us to examine the long-term effects of a nutrition intervention covering most of the first 1000-d window on PA. This is of particular importance because of the evidence that PA levels tend to remain consistent from childhood to adulthood. With regard to limitations, PA may be underestimated as a result of mispositioning of the accelerometer on the hip or removal during bathing, but caregivers were shown how to fit devices correctly after any removal and data collectors made routine visits mid-weekly to ensure correct wearing of devices. Generally, accelerometers do not differentiate between activity type (e.g. sitting, walking and running); thus, inclusion of direct observation methods in the present study may have provided complementary information on the type and context of PA. However, the logistics of the study did not allow for such observation. Nonetheless, accelerometry is a well-established technique for measuring activity in preschoolers and the ActiGraph has been validated for use in this age group^(29)^. We observed a differential loss to follow up between intervention groups, with a greater loss in the IFA group, mainly due to out-migration, which may have biased our results. However, sensitivity analysis using the multiple imputation method to impute values for missing data showed a similar pattern of results, although somewhat attenuated, suggesting that missingness of data did not introduce substantial bias. Furthermore, the baseline maternal characteristics of those included in this sub-study differed from those not included with respect to maternal age and parity, and nulliparity was associated with greater time spent in SB. This suggests that the results might not be generalisable to the full study population. Due to the high rate of attrition in our sample, our sample size was 353 participants instead of the original target number of 396, which was determined based on a power analysis for a three-group comparison. Because there were no significant differences in outcomes between the IFA and MMN groups, we combined them into a single control group and performed analyses based on a two-group comparison (LNS *v*. non-LNS), per our statistical analysis plan. With the final sample size of 353 children and the two-group comparison, we had 80 % power to detect an effect size of 0·32 SD, which was similar to the original target effect size of 0·33 SD among 396 children in the three-group comparison. Lastly, although we prespecified only one primary outcome and two secondary outcomes, we compared several secondary outcomes simultaneously in exploratory analyses and it is possible that some of our findings may be due to chance because of multiple testing^(55)^. However, these outcomes were measured at the same time and were highly correlated with each other; thus, it was logical that they would be analysed together. Under such circumstances, correcting for multiplicity may be unnecessary and counterproductive^(55)^.

In conclusion, preschool-aged children in this sub-sample who had received supplementation with LNS prenatally and from age 6 to 18 months were slightly less active and spent higher amounts in sedentary time per day compared with non-LNS children during wake time and accelerometer wear time. This finding may have been due to chance or to a difference in behavioural problems between the intervention groups, as other explanations of biological plausibility were not supported by the data or could not be explored due to lack of information. Further follow-up of this cohort should be considered to examine the long-term consequences of the low PA and relatively high percentage of time spent in SB in this sample in later childhood and adolescence. Additional research is important to understand the factors associated with SB in this population to inform intervention efforts. Since the majority of 4–6-year-old children in this population were already attending preschool for at least half a day, community-wide interventions especially through preschools or childcare programmes could be used to increase PA and to reduce lifestyle habits that promote SB in young children, to levels appropriate for positive health and development.

## Supplementary material

For supplementary material/s referred to in this article, please visit https://doi.org/10.1017/S0007114519001636

## Abbreviations:

IFA,: Fe and folic acid;
iLiNS,: International Lipid-Based Nutrient Supplements;
LNS,: lipid-based nutrient supplement;
MMN,: multiple micronutrients;
MVPA,: moderate-to-vigorous physical activity;
PA,: physical activity;
SB,: sedentary behaviour;
TV,: television;
VM,: vector magnitude.
